# Chicago Medical Society

**Published:** 1889-02

**Authors:** 


### SOCIETY REPORTS.

### CHICAGO MEDICAL SOCIETY.

*Stated Meeting, February 4.*

The President in the Chair.

Professor J. S. Knox read a paper on

ÆTIOLOGY OF PUERPERAL FEVER, in which he stated that up to 1846 our ideas as to the ætiology of puerperal fever were confused and conflicting. In 1847 Semmelweiss declared septic infection to be the cause of the disease. In 1863 Pasteur published his researches on fermentation and putrid infection. In 1866 Lister formulated his methods of antiseptic surgery, and in 1870 Stedfeld first practiced, and reported his results in, strict antiseptic midwifery. In 1846 the mortality in the maternities of Europe among lying-in women was 8 to 11 per centum. In 1883, out of 785 cases of labor there was not a single death from puerperal fever in Tarnier's Pavilion. Look at these facts as we may, and the subsequent study of them, and we can reach but one conclusion, and that is that the cause of the disease is sepsis, probably due to entrance of microbes. It is not autogenetic. Where absolute and perfect asepsis has been secured, there is not the slightest indication of puerperal fever among lying-in women. It is sometimes almost impossible to elude the subtle entrance of the microbes of sepsis, hence puerperal fever may follow as a consequence, but epidemics of the disease are entirely a thing of the past. There is no such thing to-day—where strictly antiseptic midwifery is practiced—as an epidemic of the disease. It is true, a few sporadic cases are arising here and there, but these are due to careless and improper methods of treatment.

Dr. J. C. Hoag read a paper on the *Pathology of Puerperal Fever;* Professor F. S. Johnson on the *Relation of Bacteria to Puerperal Fever;* Dr. H. P. Neuman on the *Symptoms and Diagnosis ôf Puerperal Fever*, and Professor W. W. Jaggard on the *Prevention of Puerperal Fever.*

"The Treatment of Puerperal Fever" was the title of a paper by Professor Charles W. Earle, which appears elsewhere in this number of The Journal and Examiner.

Professor R. N. Isham, in opening the discussion, said he could add but little to what had already been said. The essayists had dealt with the subject exhaustively. He was one of the few, however, that took a retrospective view of the subject. The question of the contagiousness of puerperal fever had arisen, and was denied by eminent authorities. In the light of modern pathology there was no such thing as an epidemic of puerperal fever, although there are at the present day some who entertain its existence *sui generis.* It is essentially a surgical condition, which is expressed in the words septicæmia, pyæmia, or mixed infection. The question that has arisen with reference to the matter of contagion—how far the individual practitioner is responsible for these conditions—has been ably expressed in the paper read by Professor Jaggard. The element of infection might be summed up in the axiom, *that the parturient woman is a wounded woman*, and that her condition is much like that of a person with a wound in surgical practice. The treatment is essentially the same as in other infected wounds encountered in surgical practice. A patient with puerperal fever should be regarded as other subjects of septicæmia, pyæmia, or the mixed infectious conditions. Hasty, impulsive action on part of the obstetrician in the removal of the placenta in order to get away from his case should be condemned, hence the so-called Credé's method is a great step in preventing the introduction of the hand into the uterus or uterine cavity, and the admission of air as a source of infection. In ordinary cases the parturient woman does better without an obstetrician than with him. It is the meddlesome interference in the removal of the placenta that is sometimes the cause of so many deaths.

Professor Charles T. Parkes expressed himself as being in harmony with all the methods of treatment advocated by the essayists, and believed that if the plans outlined in the papers were adopted, thoroughly and conscientiously carried out, no matter what the surroundings or condition of the patient may be, cases of puerperal fever would seldom be seen. He would indorse the surgical interference referred to by Professor Earle, but could not agree with him in the matter of interfering with the inside of the uterus, for that should be let alone, unless there were positive reasons *why* it should be interfered with. In the manifestations or sequelæ of the disease, such as decomposing fluids in the peritoneal cavity, and the presence of localized spots of suppuration, the abdominal cavity should be opened in order to get rid of the source of trouble which is causing the oncoming fatal result.

Professor D. T. Nelson said there was one drug which had not been mentioned—not believing that drugs were so over-valuable,—but it seemed to him in some of the dreadful cases of puerperal fever, when the obstetrician has nothing but drugs to rely on, among others *pilocarpine* might be used with a view to urging the skin to act in the right direction, and which had given him considerable satisfaction along with other treatment. It is not to be used alone, nor to the exclusion of the drugs that had been mentioned by Professor Earle. With reference to saline cathartics, it had been demonstrated that when salines were freely used and abundant catharsis resulted, the patient was greatly benefited, providing there was nothing in the interior of the uterus.

He had used sulphuretted hydrogen in a few instances, but not in enough of them to enable him to speak authoritatively. Knowing that it had been used in tubercular diseases, and had become more or less popular, he thought it would be of some advantage in puerperal septicæmia among other drugs that might be used. He had seen three fatal cases in consultation within the last ten days, and believed all of them were infected by midwives. How could this be prevented? The society should formulate some plan whereby all midwives should be instructed so as not to infect patients, and then compel them to follow such instructions.

Dr. J. C. Hoag referred to the lateral position in labor. It seemed to him as a distinctly prophylactic measure, because it was in this position alone that an adequate view of the perineum could be had; and as a corollary of this, it certainly enabled one to judge as to when episiotomy should be performed.

Professor Jaggard, in closing the discussion, said there was grave objection in all cases of septic poisoning to quinine, antipyrin, and the salicylates. In the first place, they did but little, if any, good in influencing the septic process, and in the second place, they invariably, to a greater or less degree, depress the heart's action. The treatment of puerperal septicæmia—so far as conducted through the alimentary canal, resolves itself practically into *milk and whiskey.*

